# Uncovering controlling factors on rock glacier velocities in the Pamir–Karakoram–Kunlun region using explainable machine learning

**DOI:** 10.1093/pnasnexus/pgag177

**Published:** 2026-05-20

**Authors:** Zhangyu Sun, Lin Liu, Tobias Bolch

**Affiliations:** Jiangsu Key Laboratory of Soil and Water Processes in Watershed, College of Geography and Remote Sensing, Hohai University, Nanjing 211100, China; Department of Earth and Environmental Sciences, Faculty of Science, The Chinese University of Hong Kong, Hong Kong 999077, China; Institute of Geodesy, Graz University of Technology, Graz 8010, Austria; Department of Earth and Environmental Sciences, Faculty of Science, The Chinese University of Hong Kong, Hong Kong 999077, China; Institute of Environment, Energy and Sustainability, The Chinese University of Hong Kong, Hong Kong 999077, China; Institute of Geodesy, Graz University of Technology, Graz 8010, Austria

**Keywords:** rock glacier velocity, control factor, machine learning, SHAP, High Mountain Asia

## Abstract

Rock glaciers are ice-debris landforms commonly found in high mountain environments. Shaped by long-term creep of ice-rich permafrost, they provide critical information for permafrost studies, mountain hydrology, and hazard assessment. Although the characteristics and controlling factors of rock glacier velocities across various temporal scales have been studied at individual sites, their environmental drivers in the spatial domain over large regions remain poorly understood. In this study, we employ four machine learning methods, i.e. support vector machine, extreme gradient boost, random forest, and backpropagation neural network, to model the relationship between rock glacier velocities and environmental variables for 5,163 rock glaciers in the Pamir–Karakoram–Kunlun region. Subsequently, we use SHapley Additive exPlanations to quantify variable importance. Results show that the upslope connection to a glacier is a critical factor controlling rock glacier velocities. In our study area, glacier-connected rock glaciers exhibit on average faster movement (median velocity = 38 cm/year) than talus-connected ones (median velocity = 28 cm/year). We also find that geomorphological properties exert stronger controls on the spatial variability of rock glacier velocities than regional climate variability. Rock glacier area and slope are identified as the second and third most important variables, with larger areas and steeper slopes associated with higher velocities. The snow cover duration ranks fourth, followed by precipitation, while air temperature shows minimal influence on velocity. Overall, these findings bridge a critical knowledge gap regarding the environmental controls on rock glacier dynamics at the regional scale, extending our understanding of rock glacier kinematics beyond site-specific investigations.

Significance statement Rock glaciers are indicators of mountain permafrost and important water resources, yet the factors controlling their movement speed in the spatial domain over large regions remain unclear. This study utilizes explainable machine learning to analyze over 5,000 rock glaciers in High Mountain Asia. We demonstrate that whether a rock glacier is connected to an upslope glacier is an important factor determining its velocity. Our results also reveal that geomorphological characteristics have a stronger impact on spatial movement patterns than regional climate conditions. These findings improve our understanding of how rock glaciers behave at large spatial scales and help clarify their role in mountain environments under changing climate conditions.

## Introduction

Rock glaciers are ice-debris landforms commonly found in high-mountain environments. Formed by gravity-driven creep of perennially frozen debris, they are characterized by morphologies of distinct frontal and lateral margins and often ridge-and-furrow surface topography (1–5). The surface movements of rock glaciers are primarily related to the permafrost creep, often primarily within a shear horizon at depth. This is a layer of frozen material with banded ice and pressurized water located at depths typically between 15 and 30 m depending on the rock glacier size and thickness (6, 7).

Monitoring rock glacier velocities is important for both scientific and practical motivations. Firstly, knowledge of rock glacier activity (intact or relict) is essential for modeling climate-related permafrost distribution (8, 9). Secondly, rock glacier velocities offer insights into mountain hydrology due to their correlation with internal ice volume (10, 11). Thirdly, fast-moving or destabilized rock glaciers could cause disasters, and thus, monitoring their velocities is critical for hazard management (3, 12). Fourthly, the variability of rock glacier velocities can serve as an indicator of climate change impact on permafrost and has been accepted as a new product of the Essential Climate Variable Permafrost in 2022 by the Global Climate Observing System (13–15).

Few studies have examined the characteristics of rock glacier velocities and their environmental drivers across multiple temporal scales at individual sites. Globally, rock glacier velocities are predominantly characterized by decadal acceleration trends (13, 16–18). In contrast, deceleration has also been observed at a few sites, possibly due to the thinning of rock glacier bodies (19). The interannual variations often exhibit synchronous patterns on a regional scale, particularly in the European Alps (18, 20). However, studies on the interannual variability in other regions worldwide are limited (15). The seasonal variations in rock glacier velocities follow a distinct rhythm, typically accelerating during the snowmelt season, peaking in late summer/early autumn, and reaching their minimum in late winter/early spring (21).

The observed temporal patterns are intricately linked to climatic and meteorological conditions. Air temperature affects rock glacier velocities across all timescales, especially at decadal and interannual timescales (16, 20, 22–24). Snow cover regime influences rock glacier velocities at interannual and seasonal timescales by serving as an insulation layer and providing water input during the melting season (21, 25). The liquid water from precipitation also contributes to the seasonal rhythm in rock glacier velocities (21, 26).

However, investigations into the environmental factors controlling rock glacier velocities across spatial domains remain limited. While Cai et al. (27) combined InSAR observations with statistical methods to model these velocities, which represents a pivotal step forward in our understanding of rock glacier kinematic mechanisms, most existing studies are still restricted to local scales (27–30) or lack comprehensive analysis (31–33). This gap is primarily due to the paucity of regional velocity datasets. Notably, Sun et al. (32) utilized Interferometric Synthetic Aperture Radar (InSAR) to produce a large-scale dataset for the Tibetan Plateau and its surrounding mountains, offering a valuable foundation for investigating controlling factors at a regional scale.

The objective of this study is to investigate the environmental factors controlling rock glacier velocities in the spatial domain over large regions and to quantify their importance. The environmental factors include rock glacier area, slope, mean annual air temperature (MAAT), average annual precipitation (AAP), and snow cover duration (SCD). We also explicitly evaluate the influence of upslope glaciers on rock glacier velocities. To achieve this, we first classify the rock glaciers based on their upslope connection units (see Materials and methods). Our analysis focuses on talus-connected and glacier-connected rock glaciers, as these two classes are dominant in the study area and provide the most relevant comparison for investigating glacial influence. We then model the relationship between rock glacier velocities and diverse environmental variables using multiple machine learning methods, including support vector machine (SVM), extreme gradient boost (XGBoost), random forest (RF), and backpropagation neural network (BPNN). Furthermore, we use SHapley Additive exPlanations (SHAP) to examine variable importance from these models. To our knowledge, this study represents the first of its kind that combines machine learning and SHAP to reveal the environmental controls on rock glacier velocities across a regional domain exceeding 10^5^ km^2^.

## Results

### Characteristics of talus- versus glacier-connected rock glaciers

**Figure 1:**
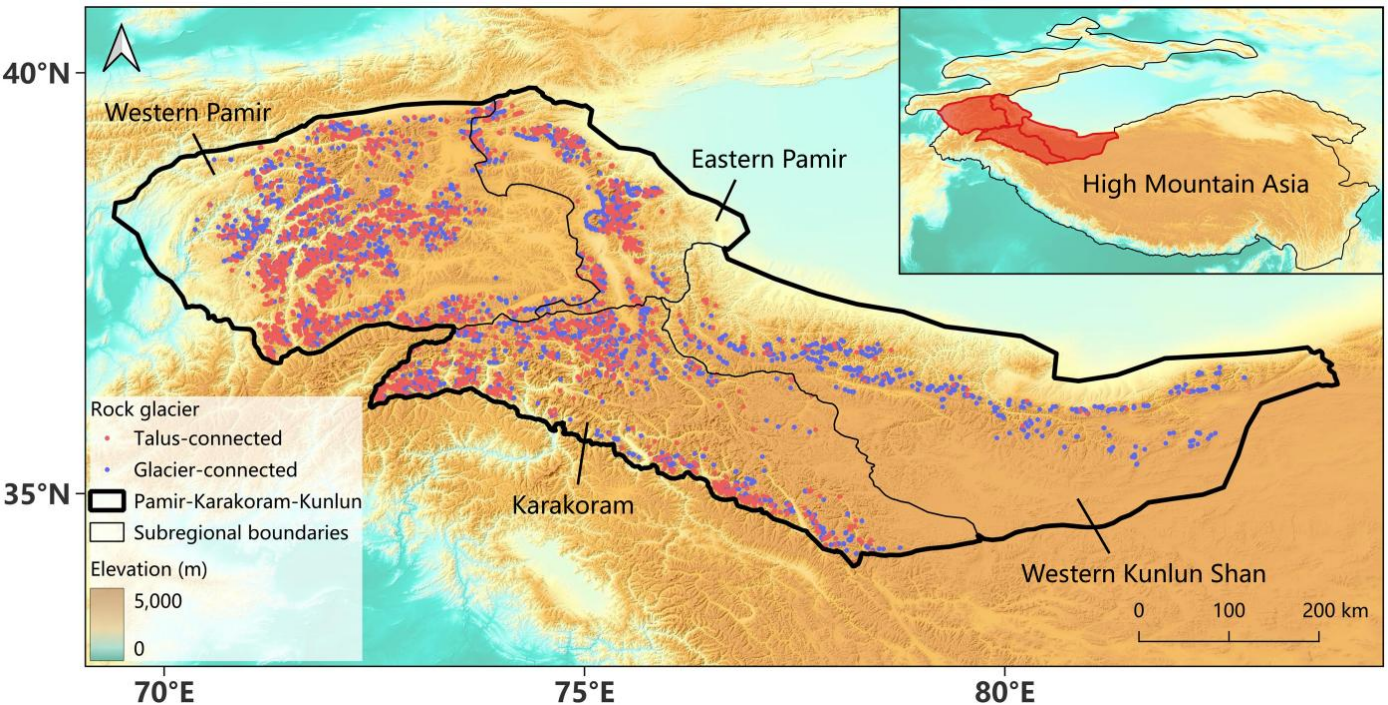
Locations of glacier- and talus-connected rock glaciers in the Pamir–Karakoram–Kunlun region, selected from the TPRoGI as compiled by (34), with areas >0.1 km^2^ and velocities between 10 and 100 cm/year as measured by (32).

**Figure 2:**
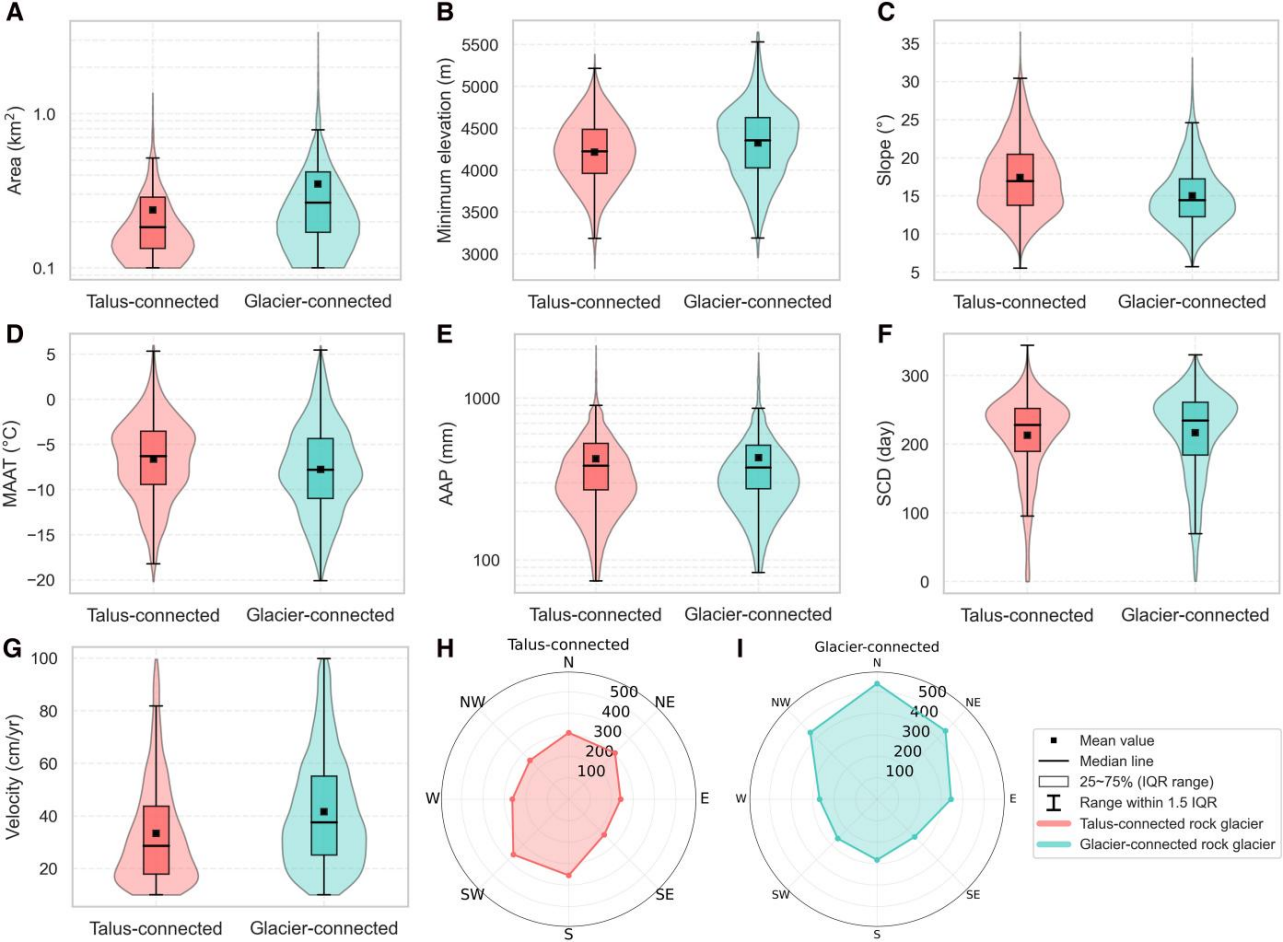
Distributions of A) rock glacier area, B) minimum elevation, C) slope, D) MAAT, E) AAP, F) SCD, and G) velocity for talus-connected and glacier-connected rock glaciers in the Pamir–Karakoram–Kunlun region. IQR, interquartile range. Panels H and I show the aspect distributions for talus- and glacier-connected rock glaciers.

We collect 5,163 rock glaciers for analysis, including 2,330 talus-connected and 2,833 glacier-connected ones (Fig. 1). Distinct discrepancies are observed in the topo-climatic characteristics between these two classes (Fig. 2). Glacier-connected rock glaciers (median area = 0.27 km^2^, interquartile range [IQR] = 0.17–0.42 km^2^) have on average larger areas than talus-connected rock glaciers (median area = 0.18 km^2^, IQR = 0.13–0.29 km^2^; Fig. 2A). The minimum elevations of glacier-connected rock glaciers (median minimum elevation = 4,357 m, IQR = 4,027–4,630 m) are slightly higher than those of talus-connected rock glaciers (median minimum elevation = 4,224 m, IQR = 3,961–4,489 m; Fig. 2B). Talus-connected rock glaciers (median slope = 17°, IQR = 14°−20°) are situated at average on steeper slopes than glacier-connected rock glaciers (median slope = 14°, IQR = 12°−17°; Fig. 2C).

Though both in cold regions, talus-connected rock glaciers (median MAAT = −6 °C, IQR = −9 to −3 °C) tend to occur in warmer regions than glacier-connected rock glaciers (median MAAT = −8 °C, IQR = −11 to −4 °C; Fig. 2D). This is due to the fact that glacier equilibrium lines in such cold environments are far above the lower limit of permafrost occurrence (35). The precipitation and snow conditions of the two classes are similar (talus-connected: median AAP = 382 mm, IQR = 272–525 mm, median SCD = 228 days, IQR = 189–252 days; glacier-connected: median AAP = 371 mm, IQR = 275–511 mm, median SCD = 234 days, IQR = 184–261 days; Fig. 2E and F).

We also find pronounced differences in rock glacier velocities. Glacier-connected rock glaciers (median velocity = 38 cm/year, IQR = 25–55 cm/year) creep on average faster than talus-connected rock glaciers (median velocity = 28 cm/year, IQR = 18–44 cm/year; Fig. 2G). This indicates that whether connected to a glacier could play an important role in rock glacier creeping rates.

Notably, we observe that the talus- and glacier-connected rock glaciers have different aspect distributions in our study area. The glacier-connected rock glaciers mainly face north, while the talus-connected rock glaciers tend to face more orientations (Fig. 2H and I). This indicates that the development of glacier-connected rock glaciers is more affected by solar radiation, whereas the occurrence of talus-connected rock glaciers is likely to be influenced by multiple factors, such as topography and climate conditions, which is likely caused by the predominant influence of radiation on the mass balance of glaciers and the microclimatic conditions of rock glaciers.

### Performance of machine learning models

**Figure 3:**
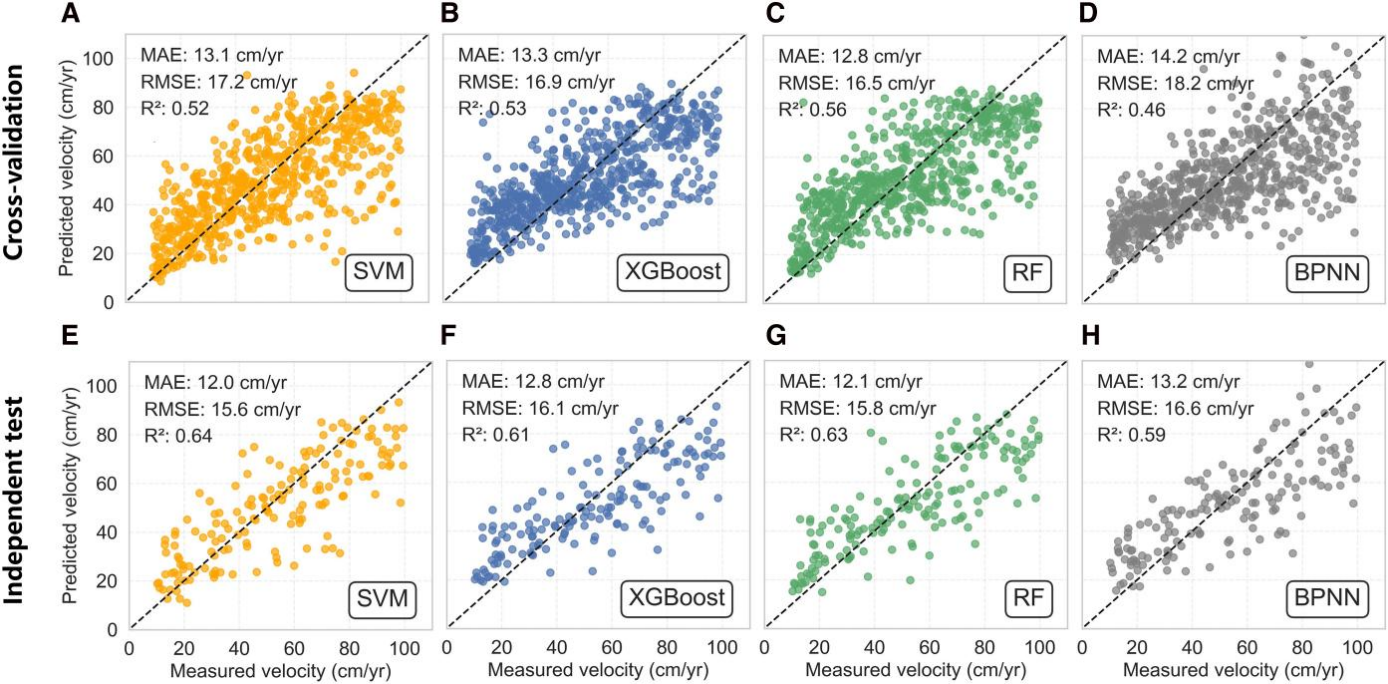
Performance of (A, E) SVM, (B, F) XGBoost, (C, G) RF, and (D, H) BPNN evaluated with (first row) cross-validation and (second row) independent test.

We compare the performance of different machine learning methods in modeling the relationship between rock glacier velocities and environmental variables. For cross-validation, RF stands out as the best model with a mean absolute error (MAE) of 12.8 cm/year, root mean squared error (RMSE) of 16.5 cm/year, and coefficient of determination (*R*^2^) of 0.56 (Table S1, Fig. 3). XGBoost and SVM are inferior to RF, achieving *R*^2^ of 0.52 and 0.53, respectively. The performance of BPNN is the worst with *R*^2^ of 0.46. For the independent test, SVM has the best performance with MAE of 12.0 cm/year, RMSE of 15.6 cm/year, and *R*^2^ of 0.64, indicating the good generalization of this model. The RF and XGBoost show relatively low performance with *R*^2^ of 0.61 and 0.64, respectively. The BPNN has the worst performance with *R*^2^ of 0.59. For both the cross-validation and independent test, BPNN has the worst performance among these four models. This may be attributed to the high performance of BPNN being dependent on a large volume of training data, and therefore, the limited number of training samples in this study restricts its performance.

### Importance of environmental variables

**Figure 4:**
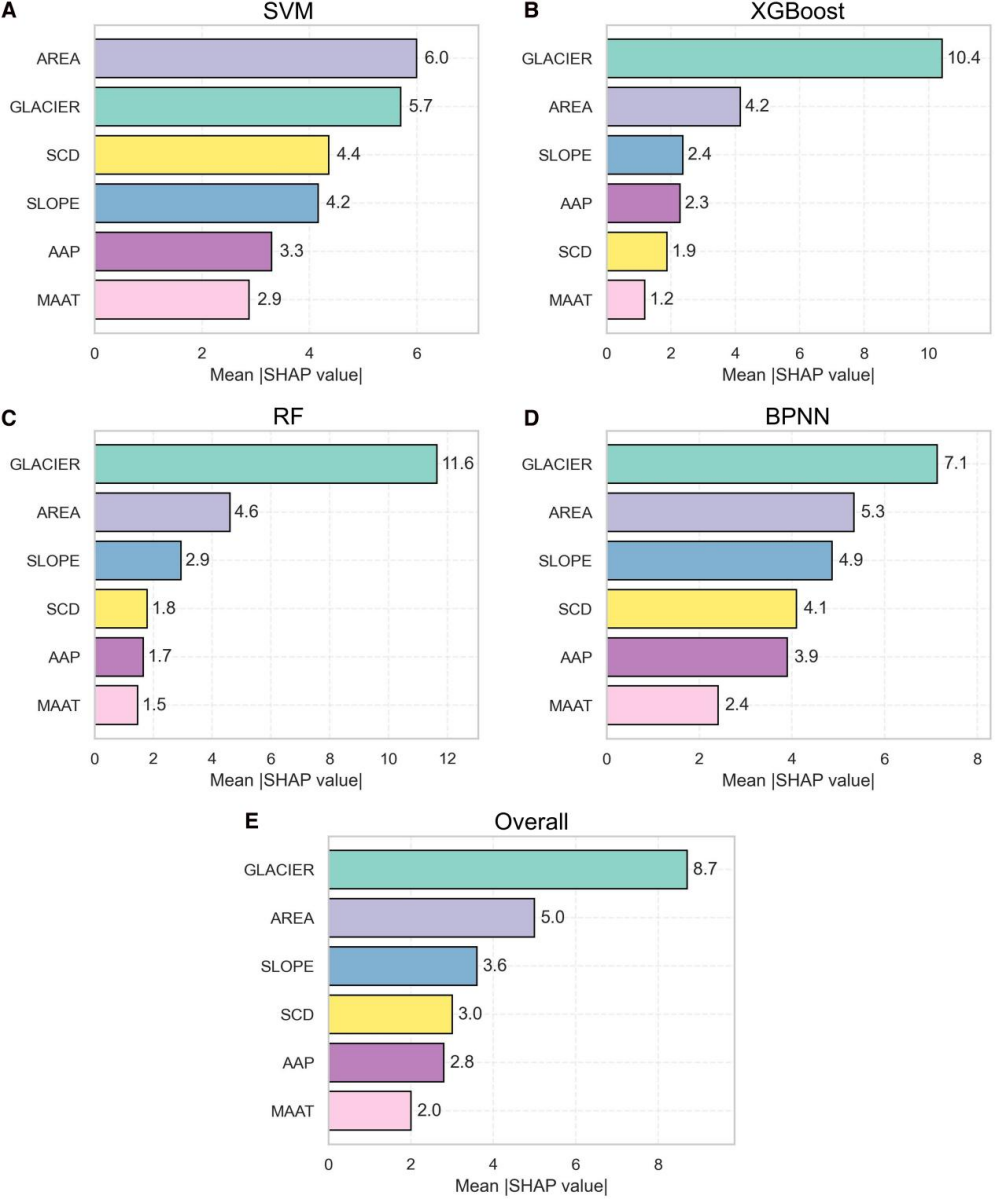
Mean absolute SHAP values of different environmental variables in explaining rock glacier velocities based on outputs from (A) SVM, (B) XGBoost, (C) RF, and (D) BPNN. (E) The overall integrated results by calculating the averaged values of the mean absolute SHAP values from the four machine learning models.

The SHAP analysis reveals that the glacier-connected factor is the most important variable explaining rock glacier velocity differences for the analyzed rock glacier types. Except for the SVM, all the other three models consistently exhibit the highest mean absolute SHAP values, with an overall average of 8.7 (Fig. 4). However, the magnitude of its impact varies between models; it is substantially higher in the RF and XGBoost models (mean absolute SHAP values >10) compared with the BPNN (mean absolute SHAP value = 7.1) and SVM models (mean absolute SHAP value = 5.7). This discrepancy shows that while the variable's top-ranked importance is consistent, the interpreted magnitude of its effect can be model dependent. Nonetheless, the consistent top ranking underscores the critical role of an upslope glacier connection in influencing rock glacier velocity.

The second most important variable is area, which generally shows moderate importance with an overall average SHAP value of 5.0. Its impact is relatively consistent across different models, with mean absolute SHAP values ranging between 4 and 6. Slope is recognized as the third important variable with an overall average SHAP value of 3.6. The magnitude of impact is more pronounced in the SVM and BPNN models, where mean absolute SHAP values exceed 4, compared with XGBoost and RF, which show lower values below 3.

SCD and AAP are the next most important factors, with the overall mean absolute SHAP values of 3.0 and 2.8, respectively. Local variability of MAAT is consistently identified as the least influential factors by all the machine learning models, and the overall mean absolute SHAP value is 2.0. This suggests that climatic factors play a less important role in explaining the observed spatial variability of rock glacier velocities than geomorphological factors in our study area.

## Discussion

### Controlling factors on rock glacier velocities at regional scale

**Figure 5:**
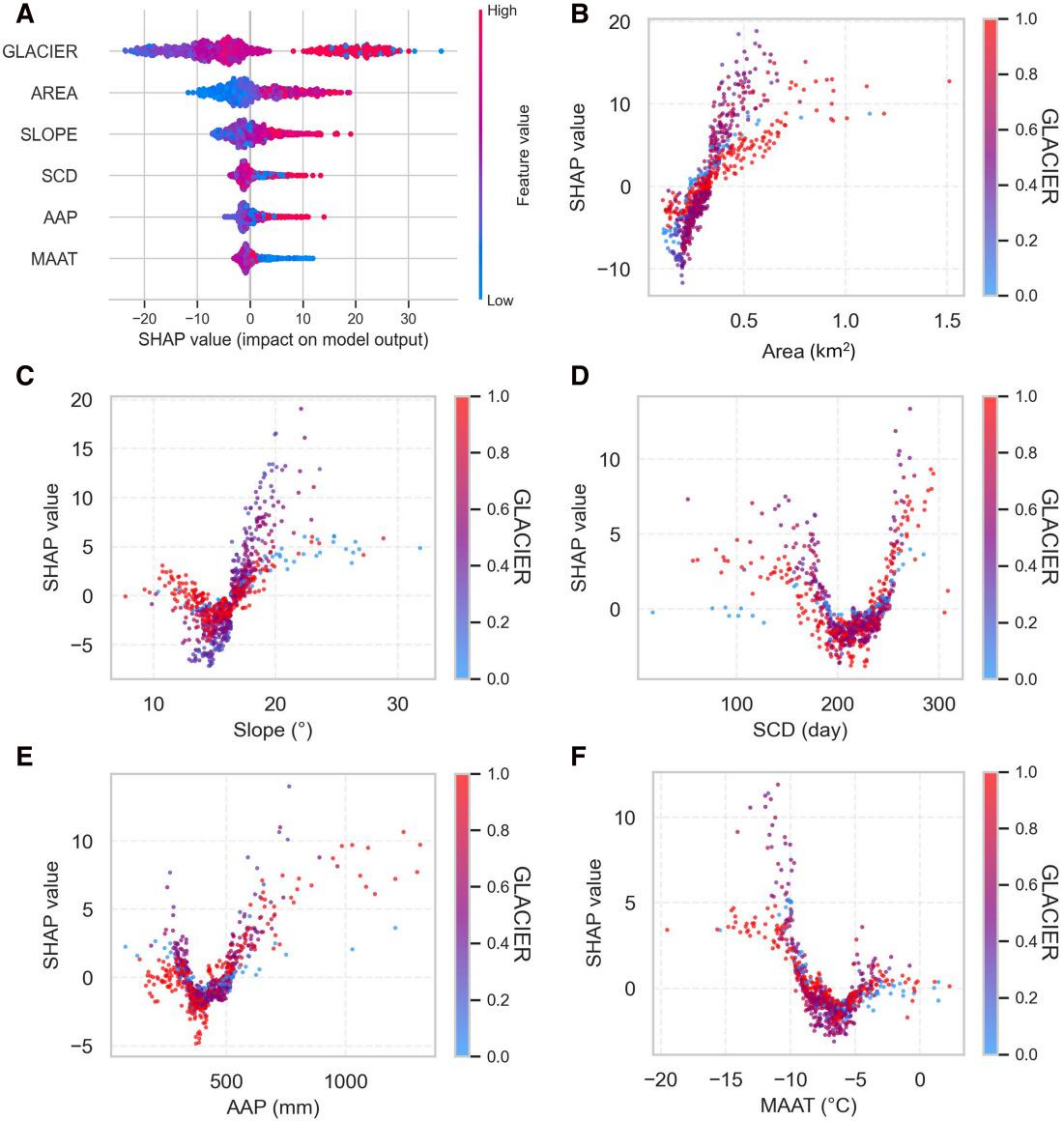
A) Beeswarm plot and dependence plots of B) area, C) slope, D) SCD, E) AAP, and F) MAAT from SHAP analysis based on RF model. The beeswarm and dependence plots based on all the machine learning models are shown in Figs. S1 and S2. SHAP values quantify the marginal contribution of each variable to the model prediction. Positive and negative SHAP values indicate the direction of the impact on rock glacier velocity, with the magnitude reflecting the relative strength of each variable's influence.

We have identified the connection to upslope glaciers as the predominant factor controlling rock glacier velocity differences between glacier- and talus-connected rock glaciers (Fig. 4). High glacier variable values are generally associated with positive SHAP values (Fig. 5A), indicating that rock glaciers connected to glaciers tend to move faster than those connected to talus. This finding aligns with the results from (36), who also reported higher velocities in glacier-connected rock glaciers in a localized study area in the southwestern Pamirs. Similarly, Zhang et al. (31) observed that rock glaciers located closer to glaciers tend to be more active. Feng et al. (37) also suggested that glaciers on the Tibetan Plateau play an important role in sustaining high deformation rates of rock glaciers. Our results provide evidence supporting the hypothesis in (32) that the contrast velocity pattern of rock glacier velocities in the westerlies- and monsoon-dominated regions on the Tibetan Plateau could be due to the disparity in the presence of rock glaciers and glaciers in these two climatic regions.

The higher activity of glacier-connected rock glaciers can be attributed to both hydrological and mechanical processes. Our study area is characterized by cold and arid conditions with glacier equilibrium line altitudes of up to 6,000 m a.s.l. (35). In this region, many glaciers are predominantly polythermal to cold-based and commonly connected to surrounding permafrost. This connectivity facilitates both hydrological continuity and mechanically stress transfer between glaciers and adjacent periglacial landforms. Firstly, sustained meltwater supply from upslope glaciers provides significant hydrological input to the rock glacier system. These water inputs infiltrate the ice-rich debris, increasing pore-water pressure and enhancing internal lubrication, which reduces deformation friction and promotes higher rock glacier velocities (38). Secondly, cold glacier margins tend to be frozen to their beds, allowing them to mechanically couple with the permafrost and transfer stress via the frozen material to the creeping rock glacier (39).

The rock glacier area is identified as the second most critical factor driving its velocity. A strong positive correlation is found between area and SHAP value, with larger areas contributing positively to predicted velocity, while smaller ones are linked to lower SHAP values (Fig. 5A). The dependence plot further illustrates this positive relationship (Fig. 5B), indicating that larger rock glaciers tend to exhibit higher velocities. Many previous studies also found similar phenomena that larger rock glaciers tend to exhibit higher velocities (29–33, 36, 37). This is likely due to the increased mass of larger rock glaciers, which results in stronger gravitational forces acting on these features and consequently leads to their higher velocities (19). Moreover, rock glacier areas likely act as a proxy for multiple underlying variables, including ice thickness, debris thickness, and landform age. For instance, larger rock glaciers may contain thicker ice bodies (7), which facilitate faster surface velocities.

Glacier-connected rock glaciers are on average larger than talus-connected ones (Fig. 2A), which could also partially explain their higher velocities. The observed area differences are likely due to their different formation processes. Glacier-connected rock glaciers could benefit from the availability of large amounts of debris material stemming from terminal moraines, while talus-connected rock glaciers typically rely on slower rockfall processes from adjacent headwalls. To quantify the influence of area on the observed velocity differences, we calculate the SHAP values for these two groups. Our analysis reveals that area contributes an average of +1.6 cm/year to the predicted velocity for glacier-connected rock glaciers, while for smaller talus-connected ones, its average contribution is −1.8 cm/year. Given the averaged velocity difference of 8.3 cm/year, the larger size of glacier-connected features could thereby explain ∼40% of the total velocity differences.

Rock glacier slope angle is identified as another significant factor, and rock glaciers occurring on steeper slopes tend to have higher velocities (Fig. 5C). This aligns with previous studies that also reported positive correlations between rock glacier velocities and slope angles (7, 27–30). This is because the steeper slope angle leads to a greater component of gravitational force parallel to the slope surface, which enhances the driving forces acting on rock glaciers and consequently increases the velocities (7). However, rock glaciers with slopes gentler than 15° can also exhibit high velocities (Fig. 5C), primarily due to the presence of glacier-connected rock glaciers with gentle slopes (indicated by red dots) that contribute to these elevated velocities. This is consistent with (29) who investigated the velocities of several large glacier-connected rock glaciers in Northern Tien Shan.

When SCD exceeds ∼220 days, a positive relationship between rock glacier velocity and SCD emerges (Fig. 5D). This threshold likely represents a transition in the ground thermal regime associated with snow insulation. Below this threshold, seasonal snow cover is relatively short-lived, facilitating efficient heat exchange between the ground surface and the atmosphere. This causes lower permafrost temperature (40), leading to slower rock glacier velocities. In contrast, when SCD exceeds the threshold, the snowpack persists throughout most of the cold season and forms a thermally efficient insulating layer. Due to its low thermal conductivity, thick and long-lasting snow cover suppresses conductive heat loss from the ground and weakens the coupling between atmospheric forcing and subsurface thermal conditions (40). As a result, the ground cools more slowly and remains at higher temperatures during winter, driving faster rock glacier movement.

Moreover, prolonged snow cover also exerts important hydrological control. Greater snow accumulation leads to increased meltwater input during the ablation season, which can elevate pore-water pressure. This process further facilitates internal deformation and basal sliding within the shear horizon, reinforcing the positive effect of SCD on rock glacier velocity.

To evaluate the consistency of this threshold, we further examine the contribution from SCD on velocities across different rock glacier types and topographic settings (Fig. S5). The threshold varies between 200 and 240 days, but a positive correlation beyond the threshold is consistently observed in most subgroups, particularly for glacier-connected rock glaciers, high-elevation (>4,000 m), and south-facing slopes, where the insulating effect of snow cover is more pronounced. In contrast, the relationship is weaker at low elevations (<4,000 m), likely reflecting the reduced influence of snow insulation and the increasing importance of other local controls.

Our findings indicate that the primary controls on rock glacier velocity differ between spatial and temporal domains. Temporally, velocity is predominantly driven by air temperature and liquid water input from snowmelt and precipitation (15, 20–22, 24, 26). Spatially, however, the dominant factors are connection to glaciers, rock glacier area, and slope. This distinction clarifies the influence of diverse environmental factors on rock glacier dynamics across different dimensions.

### Limitations and future perspectives

#### Rock glacier classification

The classification of rock glaciers based on upslope units involves interpreting complex geomorphological settings, which are often subject to subjectivity and human errors (5). These upslope units sometimes lack distinctive geomorphic features, making them easily confused with other units. For example, the ambiguity between debris-covered glaciers and glacier forefields makes the distinction between glacier-connected and glacier-forefield-connected rock glaciers challenging. Nevertheless, the distinguish between glacier-connected and talus-connected rock glaciers remains distinct in most cases. Additionally, the practical challenges in remote sensing imagery like strong snow cover frequently obscure critical morphological details, further complicating the classification process. In this study, we employ rigorous criteria and careful visual inspection with very-high-resolution satellite imagery to minimize classification errors. This robust inventory serves as a reliable foundation for the subsequent analysis. However, as highlighted by the significant influence of glaciers observed in our results, future work would benefit from a consistent and accurate inventory across the entire Tibetan Plateau that maintains this classification to allow for comparisons across different climatic domains (e.g. monsoon versus westerlies dominated).

#### Modeling constraints and data potential

The four machine learning models show differences in both predictive performance and SHAP-based feature importance. The discrepancies arise from their different algorithmic structures. Tree-based models such as RF and XGBoost are particularly effective at capturing nonlinear interactions and hierarchical relationships among variables. In contrast, SVM relies on kernel functions and may insufficiently resolve complex feature interaction. The relatively lower and less stable performance of the BPNN model is likely due to its dependence on larger training datasets.

The model performance achieves *R*^2^ between 0.52 and 0.64, indicating that ∼40% of spatial variability in rock glacier velocity remains unexplained. This is likely due to the absence of local variables such as subsurface ice content, debris thickness, lithology, and hydrological conditions. The glacier-connected variable is simplified as a binary indicator, which does not capture the degree of interaction between glaciers and rock glaciers (e.g. continuity of subsurface ice, glacier activity state, or distance to the glacier front). Moreover, the spatial resolution of the climate data (∼3 km) is coarser than the size of individual rock glaciers, which may not fully capture microclimatic variability in high mountain environments. The inherent uncertainties in InSAR-derived velocities (e.g. unwrapping errors and fixed temporal baselines) may not fully resolve the rapid velocity gradients (32). Despite these constraints, our models successfully capture key relationships between environmental variables and rock glacier velocities. To further enhance predictive power (improving upon current *R*^2^ values) and capture complex interactions, future research should integrate more variables and datasets into advanced techniques. For instance, more quantitative metrics for quantifying the interaction between glaciers and rock glaciers could better characterize glacier-rock glacier coupling. A higher-resolution dataset can better capture the microclimatic variability within local areas. Improved InSAR algorithms can mitigate phase unwrapping errors (41), while physics-informed neural networks or deep learning models (42, 43) offer a promising avenue for integrating physical laws with data-driven approaches to better model these complex interactions.

#### Spatiotemporal scale and controlling factors

Our investigation focuses on the spatial distribution of velocities at the landform level. While this approach does not resolve pixel-level heterogeneity or the full temporal evolution of velocities, it provides important insights into the broad environmental controls driving rock glacier kinematics across the region. The current analysis simplifies the complex interplay of factors by focusing on topographic and climatic variables; however, it establishes a baseline for understanding regional rock glacier dynamics. Future studies can extend this work by analyzing driving forces across both spatial and temporal scales. This would require generating regionally extensive velocity time series to assess temporal dynamics (13, 17), alongside spatial patterns. Furthermore, a more holistic mechanistic understanding could be achieved by integrating pixel-level topographic analysis and a broader suite of environmental factors, such as geological attributes (e.g. lithology) and material properties (e.g. sediment grain size and ice content).

## Materials and methods

### Study area

The study area is in the western part of High Mountain Asia, encompassing Western Pamir, Eastern Pamir, Karakoram, and Western Kunlun Shan with high elevations above 4,000 m on average and latitudes between 34°N and 40°N and longitudes between 69°E and 84°E (Fig. 1). This region is highly glacierized by hosting the major glacier systems in High Mountain Asia with ∼40,000 glaciers covering ∼40,000 km^2^ (44). According to the model from (45), permafrost covers ∼70% of the total area, occupying ∼250,000 km^2^. Approximately 10,000 rock glaciers covering 2,000 km^2^ have been identified and outlined in this region (34). Affected by westerlies, this region is characterized by a cold and dry climate with MAAT varying from −20 to 15 °C (46), AAP ranging from 40 to 800 mm with decreasing precipitation from the outer mountain ranges to the inner ones (47, 48). We select this region as the study area because it hosts one of the largest concentrations of rock glaciers in High Mountain Asia (34) and offers comprehensive velocity observations for these features (32).

### Rock glacier data

Rock glacier outlines are sourced from the Tibetan Plateau Rock Glacier Inventory (TPRoGI) as compiled by (34) using a deep learning approach followed by manual postprocessing. This inventory contains 44,273 rock glacier outlines occupying ∼6,000 km^2^ and covers an extensive area encompassing the Tibetan Plateau, and the surrounding Pamir and Karakoram mountain ranges. In the Pamir–Karakoram–Kunlun region, in total 9,716 rock glaciers were outlined in TPRoGI.

Rock glacier velocity data are obtained from (32), which employed a multitemporal multigeometry InSAR approach to generate downslope velocity fields for the rock glaciers compiled in TPRoGI using Sentinel-1 data acquired from July to August 2022. The rock glacier velocity dataset represents summer snapshot, which is expected to be about 20% higher than the annual velocities (20, 21). Since the objective of this work is to explore the control factors on rock glacier velocities on a spatial scale, it is sufficient to use a summer snapshot data. Moreover, previous studies suggest that the spatial patterns of rock glacier velocity are relatively stable across years (15), justifying this dataset's application in regional-scale spatial analysis. To account for the spatial heterogeneity of rock glacier surface movements and emphasize the active portions while excluding noise or stagnant margins, the 75th percentile of the downslope velocity field is selected to represent the velocity of each rock glacier in accordance with a previous study by (32).

### Topo-climatic data

The 30-m-resolution Copernicus GLO-30 Digital Elevation Model (49) is used to provide topographic information (elevation, slope, and aspect) for each rock glacier. The temperature and precipitation data are sourced from (46), which includes 1/30°-resolution 2-m air temperature and total precipitation monthly data from 1979 to 2023. The air temperature data are obtained by integrating short-term high-resolution Weather Research and Forecasting simulations, long-term ERA5 data, and station observation data (50). The precipitation data are from TPHiPr, a high-precision 1/30°-resolution dataset obtained by merging downscaled ERA5 products with over 9,000 rain gauge measurements using machine learning (48). The snow data are sourced from (51), which provides 500-m resolution global mountain snow cover phenology for the period 2000–2022 generated using MODIS snow products.

### Selection and classification of rock glaciers

To ensure the reliability of our analysis, we perform a quality selection on the InSAR-derived velocity dataset. According to (32), the InSAR-derived velocities for small (area <0.1 km^2^), slow-moving (velocity <10 cm/year), or fast-moving (velocity >100 cm/year) rock glaciers could have large uncertainties due to the inherent limitations of radar interferometry and the use of Sentinel-1 data with a temporal baseline of 12 days. Consequently, we select 6,178 rock glaciers with areas >0.1 km^2^ and velocities between 10 and 100 cm/year for initial analysis.

Previous studies have suggested that upslope geomorphological units, particularly glaciers, can influence the velocities of downslope-connected rock glaciers (31, 32, 37, 52). To explicitly investigate the impacts of glacier connection on rock glacier movement, we classify the rock glaciers based on their connected upslope units. According to the latest guidelines released by the International Permafrost Association Rock Glacier Inventory and Kinematics initiative, the recommended classes include talus connected, debris-mantled slope connected, landslide connected, glacier connected, glacier forefield connected, poly connected, and other (4, 5).

This analysis focuses on talus- and glacier-connected rock glaciers, as these are the predominant classes in the study area and provide the most robust basis for investigating glacial influence. A talus-connected rock glacier typically forms as part of a downslope geomorphic system composed of a headwall, an intervening talus slope, and the rock glacier body (Fig. S4A). Material supplied from the headwall accumulates on the talus slope and subsequently feeds the rock glacier downslope (4). In contrast, a glacier-connected rock glacier evolves as an extension of a glacier, debris-covered glacier, the terminal moraine of a glacier, or perennial ice patch (Fig. S4B). Between the clean glacier ice and the rock glacier, a transition zone of subsurface ice is often present, characterized by geomorphological indicators such as crevasses, thermokarst depressions, and meltwater channels (4, 39). The glacier outlines from the Randolph Glacier Inventory (RGI v7.0, 43) are used to assist in classifying glacier-connected rock glaciers. The classification is conducted manually using very-high-resolution (0.5 m) Google Earth imagery and Bing Maps. Consequently, 2,330 talus-connected and 2,833 glacier-connected (in total, 5,163) rock glaciers are selected for analysis. The remaining 1,015 rock glaciers classified as other types (e.g. debris-mantled slope connected and glacier forefield connected) are excluded.

### Preparation of modeling dataset

The response variable is rock glacier velocity. We select rock glacier area, slope, MAAT, AAP, SCD, and binary glacier-connected factor as explanatory variables (Table S2). These variables represent the primary topographic and climatic controls on rock glacier kinematics. Rock glacier area and slope influence the internal deformation and gravitational driving stress. MAAT, AAP, and SCD characterize the regional thermal and hydrological conditions, which jointly govern ground ice preservation. We do not consider the rock glacier aspect, as the velocities were derived using InSAR, which is highly sensitive to aspect orientation (the InSAR-derived velocities have large uncertainties for the north- and south-facing rock glaciers).

Prior to the machine learning modeling, we perform a data balancing procedure. This is because the dataset is imbalanced, with most rock glaciers exhibiting slow movements. This may bias the model toward predicting slow velocities while performing poorly on fast-moving cases (53). To ensure that the training samples are evenly distributed, we group the rock glaciers into 0.1 cm/year velocity bins and average the velocities and environmental variables within each bin. This approach serves two purposes: (i) to mitigate data imbalance by ensuring a more uniform distribution of samples across velocity ranges and (ii) to reduce noise associated with InSAR measurements at individual rock glaciers by averaging values within bins (54). The choice of bin size is critical: a wide bin may oversimplify the dataset by losing important variability, whereas a narrow bin may fail to effectively balance the data. We test bin sizes ranging from 0.001 to 1 cm/year and select 0.1 cm/year as it provides an optimal balance between data diversity and model performance (Table S3). Consequently, we collect 814 samples for developing machine learning models.

The Pearson correlation analysis shows that the correlation coefficients between all the environmental variables are <0.7 (Fig. S5), indicating that the multicollinearity is not present in the dataset (55). Relatively high-positive correlations can be found between velocity and area (*R* = 0.35) and glacier-connected factor (*R* = 0.32), suggesting the importance of these two factors on influencing rock glacier velocities.

### Machine learning modeling

We select four machine learning models to identify the controlling factors on rock glacier velocities from diverse machine learning families, including the kernel-based SVM, the tree-based RF and XGBoost, and the neural network BPNN. SVM is a maximum-margin classifier that finds the optimal hyperplane to separate data by class. The kernel function allows for efficient and nonlinear separation by implicitly mapping data into high-dimensional feature spaces (56). RF is an ensemble method that combines bagging and random feature selection to construct decorrelated decision trees. By aggregating their predictions, it significantly reduces variance and overfitting, resulting in a robust and interpretable model (57). XGBoost is a scalable gradient boosting framework that sequentially builds an ensemble of weak models, with each new model correcting the errors of its predecessors. Its efficiency and superiority stem from its regularized model objective and sparsity handling (58). BPNN is a hierarchical model composed of layers of interconnected neurons. Through backpropagation, it learns complex and hierarchical feature representations by capturing intricate patterns in high-dimensional spaces (59).

We use an 8:2 ratio to split all the samples into training and test datasets. The training datasets are first used to optimize hyperparameters using a 5-fold cross-validation grid search approach (Table S4). Then, all the training datasets are used to fit a model using the optimal hyperparameters. The test datasets do not participate in the training and hyperparameter optimization processes, which serve as independent data to evaluate model generalization. This approach comprehensively evaluates model performance by providing both cross-validation evaluation and independent test results.

We employ three accuracy metrics to evaluate model performance, including MAE, RMSE, and *R*^2^. MAE measures average error magnitude while RMSE emphasizes larger errors due to its quadratic nature. Lower MAE and RMSE indicate better performance. *R*^2^ quantifies the goodness-of-fit, with a maximum value of 1.0 indicating a perfect fit. The equations for calculating these metrics are as follows:


(1) $$\mathrm{MAE}=\frac{1}{n}\overset{n}{\underset{i=1}{\sum }}\;|y_{i}-{\hat{y}}_{i}|$$

(2) $$\mathrm{RMSE}=\sqrt{\frac{1}{n}\overset{n}{\underset{i=1}{\sum }}\;{\left(y_{i}-{\hat{y}}_{i}\right)}^{2}}$$

(3) $$R^{2}=1-\frac{\sum_{i=1}^{n}\;{\left(y_{i}-{\hat{y}}_{i}\right)}^{2}}{\sum_{i=1}^{n}\;{\left(y_{i}-\bar{y}\right)}^{2}}$$

where *n* is the number of observations, $y_{i}$ is the actual value, ${\hat{y}}_{i}$ is the predicted value, and $\bar{y}$ is the mean of the actual value.

### SHAP interpretation

The outputs of the four machine learning models from all samples are fed into SHAP to quantify the importance of the environmental variables in explaining rock glacier velocities. SHAP is a unified framework for interpreting machine learning model predictions based on game theory (60). It uses Shapley values to quantify the contributions of each variable on model predictions.

We first calculate the SHAP values for all samples, which are then used to compute the mean absolute SHAP values to quantify the global importance of each environmental variable. To comprehensively evaluate the importance, we integrate the results by calculating the averaged values of the mean absolute SHAP values from the four machine learning models. We also analyze the dependence of rock glacier velocities on each environmental variable.

## Supplementary Material

pgag177_Supplementary_Data

## Data Availability

The rock glacier outline data are available at https://doi.org/10.5281/zenodo.10732042. The rock glacier velocity data are available at https://doi.org/10.5281/zenodo.15847163. The air temperature and precipitation data are available at https://doi.org/10.11888/Atmos.tpdc.300398. The snow cover duration data are available at https://doi.org/10.48784/1zvv-nw59. The data and codes used in this study are provided via the zenodo open-access repository at https://doi.org/10.5281/zenodo.18195131.
