# Supplementary material for: Uncovering controlling factors on rock glacier velocities in the Pamir–Karakoram–Kunlun region using explainable machine learning
Source: PNAS Nexus. 2026 May 20;5(5):pgag177. doi: 10.1093/pnasnexus/pgag177 (PMC13220748; doi:10.1093/pnasnexus/pgag177)
Supplement: pgag177_Supplementary_Data [file pgag177_supplementary_data.docx]

**
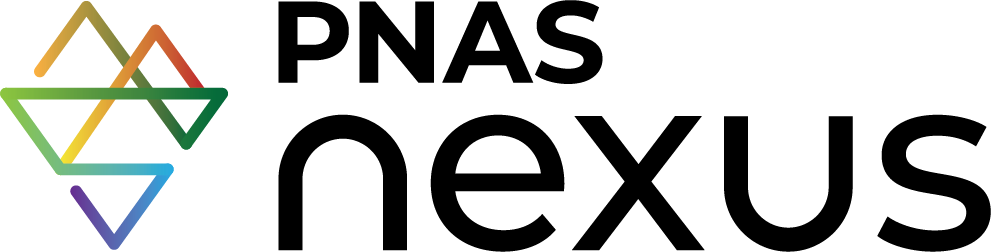
**

**Supplementary Information for**

Uncovering controlling factors on rock glacier velocities in the Pamir-Karakoram-Kunlun region using explainable machine learning

Zhangyu Sun, Lin Liu, and Tobias Bolch

Zhangyu Sun

Email: sunzhangyu@link.cuhk.edu.hk

**This PDF file includes:**

Figures S1 to S5

Table S1 to S4


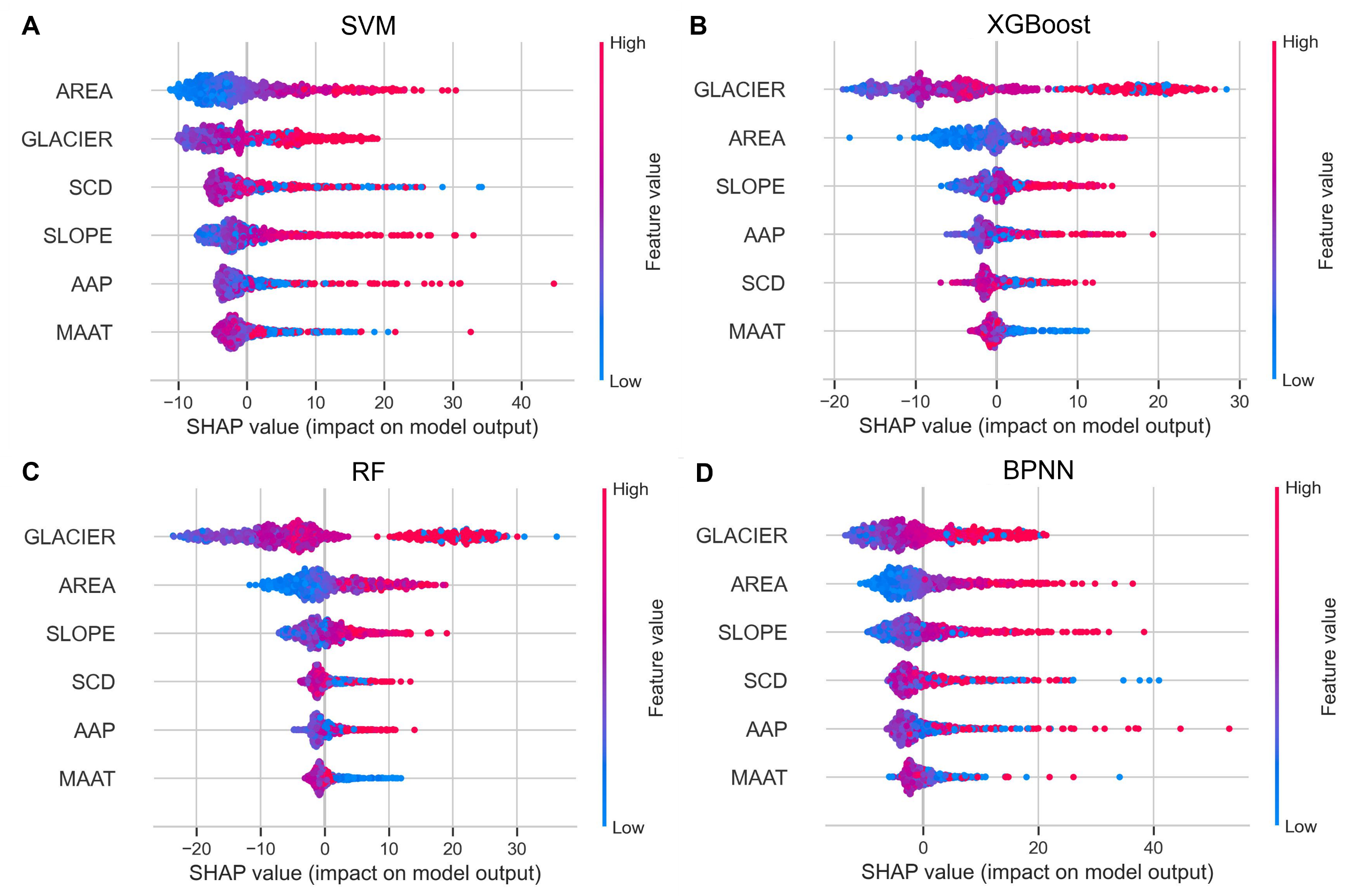


**Fig. S1.** Summary beeswarm plots of (A) Support Vector Machine (SVM), (B) Extreme Gradient Boost (XGBoost), (C) Random Forest (RF), and (D) Back Propagation Neural Network (BPNN).


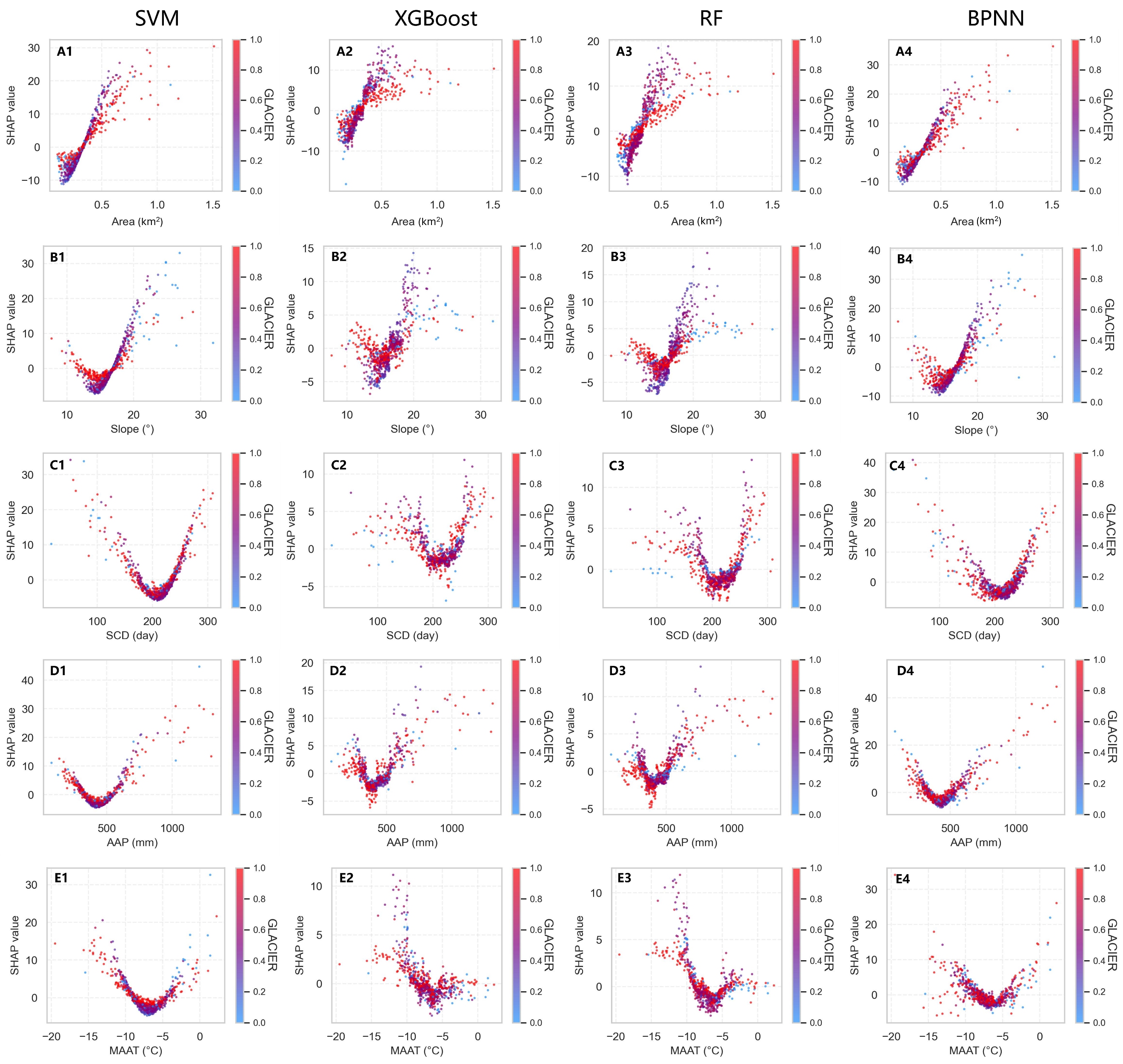


**Fig. S2.** Dependence plots of each environmental variables for (first column) SVM, (second column) XGBoost, (third column) RF, and (fourth column) BPNN.


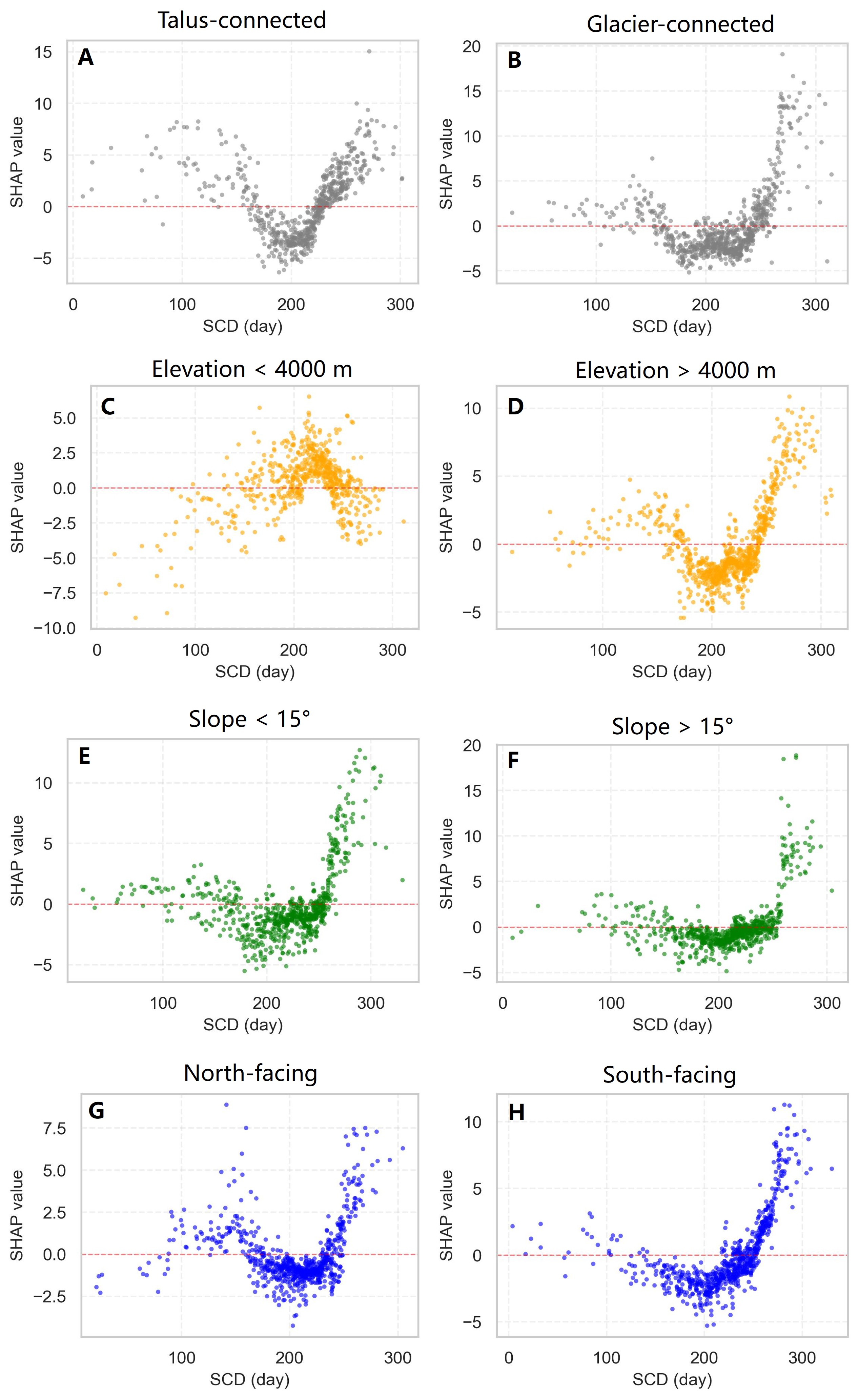


**Fig. S3.** Dependence plots of SCD from SHAP analysis based on RF model in different rock glacier types and topographic settings: (A) talus-connected, (B) glacier-connected, (C) elevation < 4000 m, (D) elevation > 4000 m, (e) slope < 15°, (F) slope > 15°, (G) north-facing, (H) south-facing.


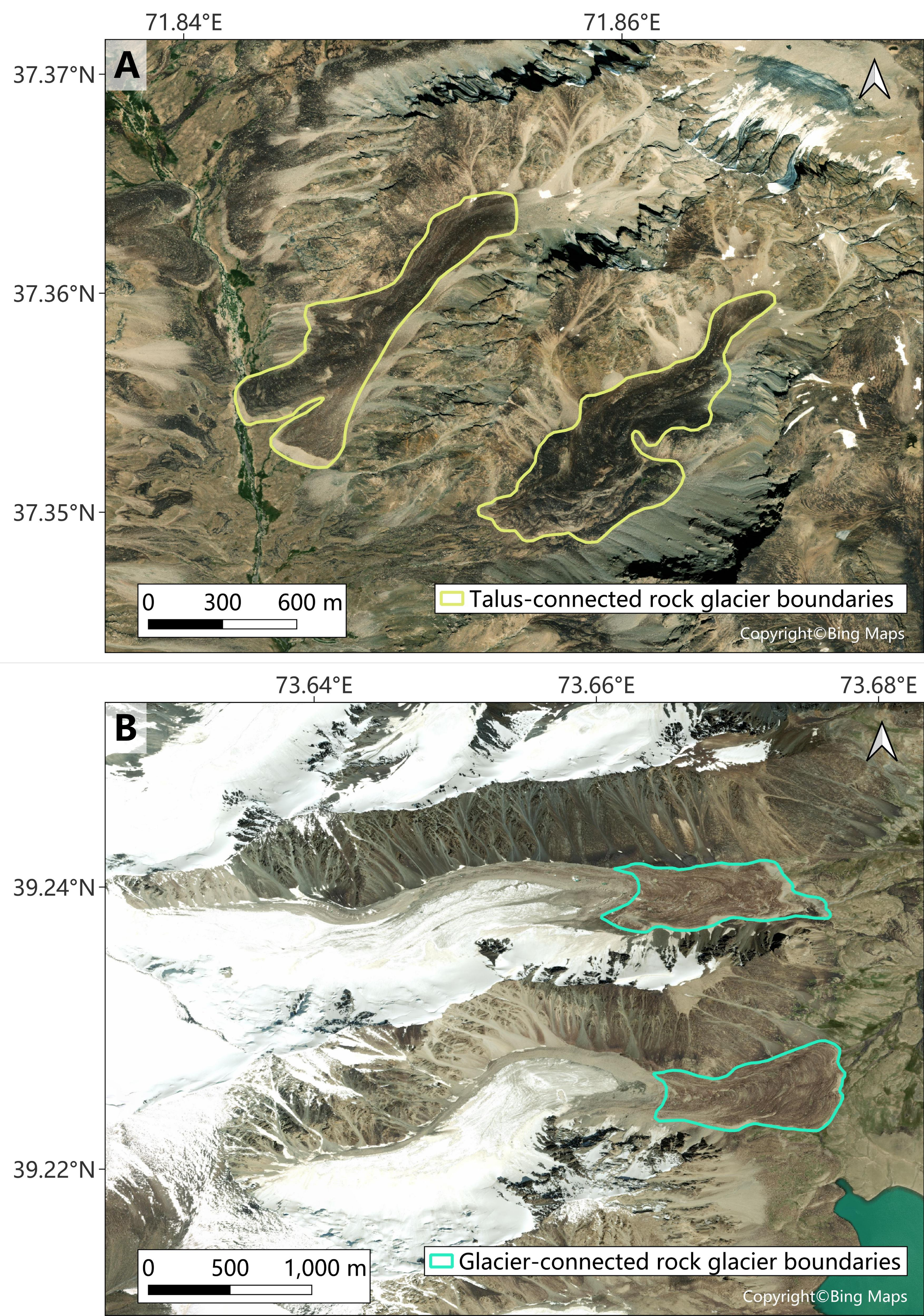


**Fig. S4.** Visual examples of (A) talus-connected rock glaciers and (B) glacier-connected rock glaciers.


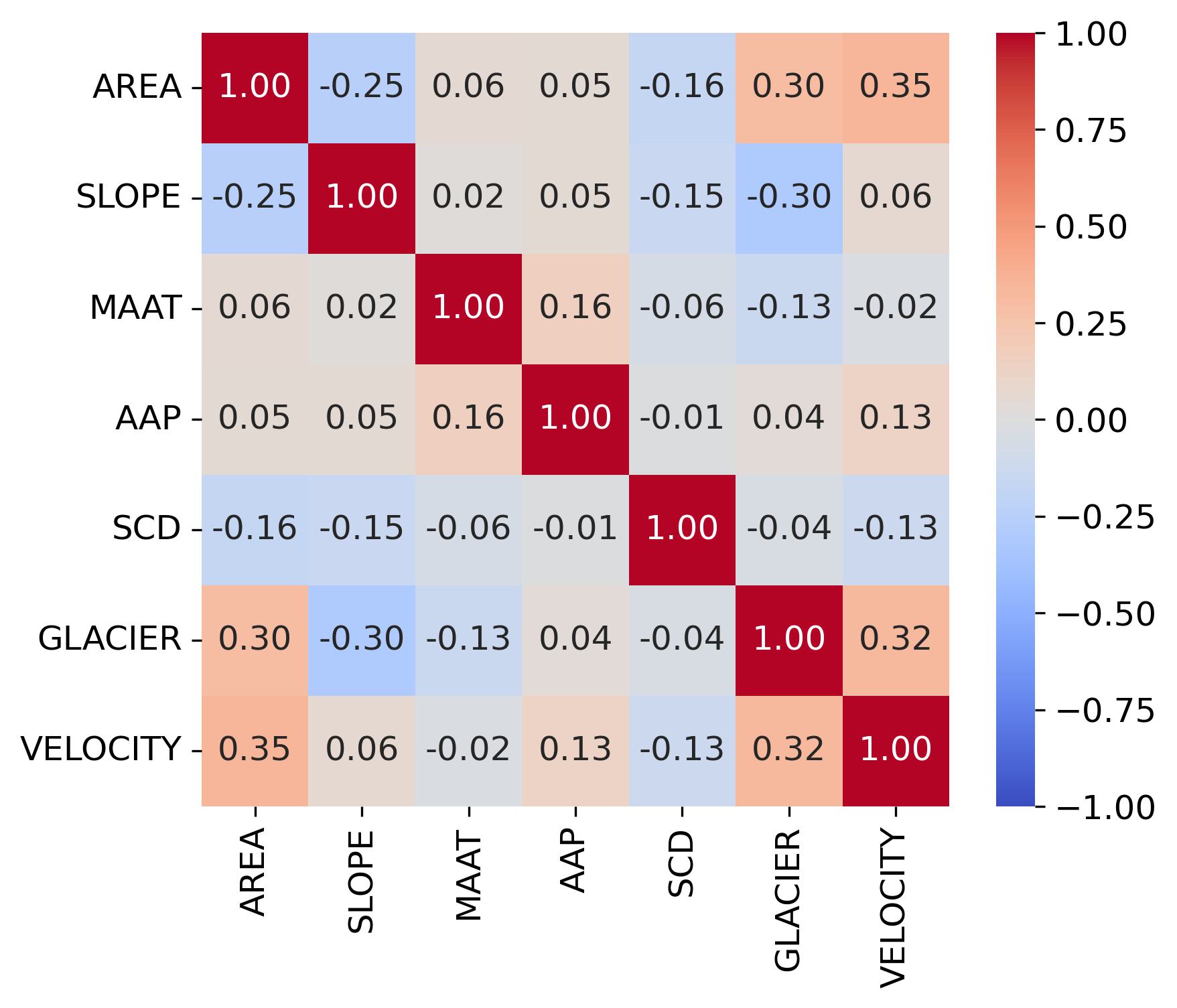


**Fig. S5.** Pearson correlation coefficients between environmental variables and rock glacier velocity.

**Table S1.** Mean Absolute Error (MAE), Root Mean Squared Error (RMSE), and Coefficient of Determination (R^2^) of different machine learning models.

| Machine learning model | Cross-validation | | |  | Independent test | | |
| --- | --- | --- | --- | --- | --- | --- | --- |
|  | MAE (cm/yr) | RMSE (cm/yr) | R^2^ |  | MAE (cm/yr) | RMSE (cm/yr) | R^2^ |
| SVM | 13.1 | 17.2 | 0.52 |  | **12.0** | **15.6** | **0.64** |
| XGBoost | 13.3 | 16.9 | 0.53 |  | 12.8 | 16.1 | 0.61 |
| RF | **12.8** | **16.5** | **0.56** |  | 12.1 | 15.8 | 0.63 |
| BPNN | 14.2 | 18.2 | 0.46 |  | 13.2 | 16.6 | 0.59 |

**Table S2.** Explanatory and response variables and data sources.

| Variable | Description | Units | Data source | Resolution | Reference |
| --- | --- | --- | --- | --- | --- |
| AREA | Rock glacier area | m^2^ | TPRoGI | - | Sun et al., 2024 |
| SLOPE | Median slope | ° | Copernicus DEM | 30 m | European Space Agency, 2024 |
| MAAT | Mean Annual Air Temperature | °C | TPMFD | 1/30° | Yang et al., 2023 |
| AAP | Average Annual Precipitation | mm | TPHiPr | 1/30° | Jiang et al., 2023 |
| SCD | Snow Cover Duration | day | MODIS snow products | 500 m | Notarnicola, 2020 |
| GLACIER | Glacier-connected factor (1 for glacier-connected, 0 for talus-connected) | - | Manual classification | - | This study |
| VELOCITY | Rock glacier velocity | cm/yr | InSAR measurement | - | Sun et al., 2025 |

**Table S3.** Sample size and XGBoost model performance in different bin sizes of rock glacier velocities

| Bin size (m/yr) | Sample size | Cross-validation R^2^ | Independent test R^2^ |
| --- | --- | --- | --- |
| 1 | 90 | 0.56 | 0.75 |
| 0.1 | 814 | 0.53 | 0.61 |
| 0.01 | 3520 | 0.15 | 0.18 |
| 0.001 | 4942 | 0.13 | 0.11 |

**Table S4.** Optimized hyperparameters of machine learning models. Hyperparameters are optimized using a grid search approach combined with five-fold cross-validation.

| Machine learning  model | Model parameter | Hyperparameters for grid search | Optimal hyperparameter |
| --- | --- | --- | --- |
| SVM | C | [0.1, 1, 10, 100] | 10 |
|  | Kernel | rbf | rbf |
|  | gamma | scale | scale |
| XGBoost | n_estimators | [20, 50, 100, 200, 300] | 300 |
|  | max_depth | [3, 5, 7, 10] | 5 |
|  | learning_rate | [0.001, 0.01, 0.1, 0.2] | 0.01 |
| RF | n_estimators | [20, 50, 100, 200, 300] | 200 |
|  | max_depth | [5, 10, 20, 30] | 10 |
|  | min_samples_split | [2, 5, 10] | 10 |
|  | min_samples_leaf | [1, 2, 4] | 1 |
| BPNN | hidden_layer_sizes | [8, 16, 32, 64, 128, 256] | 64 |
|  | activation | relu | relu |
|  | solver | adam | adam |
|  | alpha | [0.0001, 0.001, 0.01] | 0.01 |
|  | learning_rate_init | [0.001, 0.01, 0.1] | 0.1 |
|  | max_iter | [500, 1000, 2000] | 500 |
